# Evaluating the quality, safety, and functionality of commonly used smartphone apps for bipolar disorder mood and sleep self-management

**DOI:** 10.1186/s40345-022-00256-6

**Published:** 2022-04-04

**Authors:** Emma Morton, Jennifer Nicholas, Linda Yang, Laura Lapadat, Steven J. Barnes, Martin D. Provencher, Colin Depp, Michelle Chan, Rhea Kulur, Erin E. Michalak

**Affiliations:** 1 Department of Psychiatry, University of British Columbia, 420-5950 University Boulevard, Vancouver, BC V6T 1Z3 Canada; 2 Orygen, Parkville, VIC Australia; 3 Centre for Youth Mental Health, University of Melbourne, VIC, Australia; 4 Department of Psychology, University of British Columbia, Vancouver, BC Canada; 5 École de Psychologie, Université Laval, Québec, QC Canada; 6 Department of Psychiatry, University of California, San Diego, CA USA

**Keywords:** mHealth, Bipolar disorder, Self-management, Apps, Self-monitoring

## Abstract

**Background:**

Individuals with bipolar disorder (BD) are increasingly turning to smartphone applications (apps) for health information and self-management support. While reviews have raised concerns regarding the effectiveness and safety of publicly available apps for BD, apps surveyed may not reflect what individuals with BD are using. The present study had two aims: first, to characterize the use of health apps to support mood and sleep amongst people with BD, and second, to evaluate the quality, safety and functionality of the most commonly used self-management apps.

**Methods:**

A web-based survey was conducted to explore which apps people with BD reported using to support self-management of mood and sleep. The characteristics of the most commonly nominated apps were described using a standardized framework, including their privacy policy, clinical foundations, and functionality.

**Results:**

Respondents (*n* = 919) were 77.9% female with a mean age of 36.9 years. 41.6% of participants (*n* = 382) reported using a self-management app to support mood or sleep. 110 unique apps were nominated in relation to mood, and 104 unique apps nominated in relation to sleep; however, most apps were only mentioned once. The nine most frequently nominated apps related to mood and sleep were subject to further evaluation. All reviewed apps offered a privacy policy, however user control over data was limited and the complexity of privacy policies was high. Only one app was developed for BD populations. Half of reviewed apps had published peer-reviewed evidence to support their claims of efficacy, but little research was specific to BD.

**Conclusion:**

Findings illustrate the potential of smartphone apps to increase the reach of psychosocial interventions amongst people with BD. Apps were largely created by commercial developers and designed for the general population, highlighting a gap in the development and dissemination of evidence-informed apps for BD. There may be risks in using generic health apps for BD self-management; clinicians should enquire about patients’ app use to foster conversations about their particular benefits and limitations.

**Supplementary Information:**

The online version contains supplementary material available at 10.1186/s40345-022-00256-6.

## Background

Bipolar disorder (BD) is a chronic, severe mental disorder characterized by the experience of episodes of clinically depressed or elevated mood states. The lifetime prevalence of bipolar spectrum disorders is estimated to be 2.4% globally (Merikangas et al. 2011), and the condition is ranked as the 5th leading cause of disease burden among mental disorders by the World Health Organization (Ferrari et al. 2016). Self-management is recognized as central to living well with BD by both treatment guidelines (Kendall et al. 2016; Malhi et al. 2020; Yatham et al. 2018) and people living with the disorder (Michalak et al. 2016; Murray et al. 2011). Particular emphasis is placed on detecting and responding to changes in two symptom domains, mood and sleep, as there is evidence that psychosocial therapies which incorporate these strategies can reduce symptoms and prevent relapse/recurrence (Miklowitz and Scott 2009). However, distance, cost, stigma, and trust in the healthcare system present barriers to accessing clinician support for self-management practices (Leitan et al. 2015).

Digital health interventions, particularly smartphone applications (apps; otherwise known as mHealth), may improve access to evidence-based psychosocial strategies for BD. There are a number of promising developments in mHealth research for BD (Torous and Brady 2020), such as employing smartphone passive data-sensing capabilities (Faurholt-Jepsen et al. 2013; Matthews et al. 2016; Beiwinkel et al. 2016), facilitating clinician review of self-monitoring data (Faurholt-Jepsen et al. 2019), and delivering psychoeducation programs (Hidalgo-Mazzei et al. 2016; Depp et al. 2015). Despite such remarkable progress in leveraging new technologies to better monitor BD symptoms and deliver interventions, evidence-supported apps are slow to be made publicly accessible due to research-to-practice translational lags and complex regulatory systems (Mathews et al. 2019). Further, research grants rarely cover the costs required for ongoing customer/technical support, and as a consequence many academic mHealth projects do not reach commercial marketplaces (Jake-Schoffman et al. 2017). In contrast to the slow pace of development, evaluation and dissemination in academic contexts, an increasingly large number of apps claiming to support mental health needs are available for download via commercial marketplaces (Torous et al. 2018). Given that internet-connected device ownership and interest in smartphone-based interventions is high amongst people living with BD (Hidalgo-Mazzei et al. 2019), it is likely that consumers with BD are already self-selecting from available mental health apps. Accordingly, there is widespread concern about the quality, safety and efficacy of apps used by consumers (Torous et al. 2018; Larsen et al. 2019; Wisniewski et al. 2019), as individuals may be downloading and using mental health apps that lack evidence bases and without consulting their healthcare provider.

Regular review of consumer-facing apps is therefore critical for both (a) informing clinicians and individuals about the potential benefits and risks of the current mental health app marketplace, and (b) helping researchers and developers optimize the next generation of mHealth interventions by identifying gaps in the content, functionality, quality, and safety of current apps. To date, two reviews have identified and evaluated apps developed for BD. Nicholas et al. identified 82 apps developed for BD in the Google Play and Apple iOS stores (Nicholas et al. 2015). Concerningly, only 22% provided a privacy policy, and app content was largely not in line with guidelines for BD treatment, with some apps containing harmful or misleading content. More recently, a review of the top-returned apps for the keyword “bipolar” echoed these findings (Lagan et al. 2020a). Only one app had peer-reviewed literature to support its efficacy, a number of apps included harmful, stigmatizing or misleading content, and 68% of apps provided privacy policies, indicating negligible improvements in quality in the five years since the review by Nicholas et al. Other reviews have evaluated mental health apps more broadly, concluding that the quality of evidence supporting their efficacy is insufficient (Larsen et al. 2019; Wisniewski et al. 2019), and privacy policies (if existent) lack transparency (Robillard et al. 2019; Huckvale et al. 2019).

Although such reviews have elucidated the type and quality of publicly available mental health apps (including those marketed for BD), it has been noted that marketplace searches alone may not reflect the apps used in real-world contexts (Rubanovich et al. 2017). Despite the proliferation of health apps, the majority of downloads are accounted for by a small number of apps (Aitken and Lyle 2015), and download metrics may not reflect longer-term uptake. Search results are personalized based on user characteristics (Grundy et al. 2016), and popularity metrics may be biased by developers paying for downloads/positive reviews (BinDhim et al. 2015). Indeed, it has been noted that the most downloaded mental health apps are not necessarily the ones with the most active users (Carlo et al. 2020).

Conclusions drawn on the basis of marketplace searches may therefore not accurately represent real-world use. For example, while a recent review of apps for depression or anxiety reported that evidence-supported treatment components for these conditions were poorly represented (Wasil et al. 2019), when the appraisal was weighted by the number of active users, the reach of specific treatment elements changed. Although mindfulness exercises were present in only 37% of reviewed apps, this technique was encountered by the largest proportion (96%) of monthly active users. While this analysis better depicts naturalistic usage, it has limitations in representing apps used by those with mental health needs. First, user counts included downloads by healthy adults. Second, apps were identified by searching for “depression” and “anxiety”; yet people with mental health needs have reported using wellbeing apps designed for the general population, not necessarily those targeting their diagnosed conditions (Rubanovich et al. 2017; Beard et al. 2019). To accurately appraise the quality of apps adopted by people with BD in real-world contexts, we must directly ask the individuals in question to describe their mHealth use (Rubanovich et al. 2017).

The present survey aimed to identify apps used by people with BD to self-manage two core symptom domains (mood and sleep), and to evaluate their quality and features according to a standardized framework (Lagan et al. 2020b).

## Methods

### Study design

An overarching, international, cross-sectional online survey investigated the use of smartphone apps to support mental health and quality of life, attitudes towards app features, privacy concerns, and digital health literacy amongst people living with BD. The survey was conducted according to a community-based participatory research framework: the Collaborative RESearch Team to study psychosocial issues in Bipolar Disorder (CREST.BD) research network community advisory group (comprised primarily of people living with BD) was consulted regularly regarding survey content and recruitment strategies. This community-based participatory research process is described in full elsewhere (Morton et al. 2021a). Prior reports have summarized predictors of digital health literacy (Morton et al. 2022) and endorsement of various app features/privacy concerns in this sample (Morton et al. 2021a). Here, to characterize use of self-management apps specifically, we detail responses to survey items related to apps used in self-management of mood and sleep.

Questionnaires were administered via Qualtrics. Data collection occurred between February and July 2020. Ethics approval was granted by the University of British Columbia Behavioral Research Ethics Board. All participants received written information about the study and indicated their consent before proceeding. Participants who exited the survey prior to completion were treated as having withdrawn consent, and their data were not analyzed. Data in the study were treated confidentially and survey responses stored on a secure server in Canada.

### Participants and recruitment

Participant recruitment occurred via promotion on CREST.BD social media pages, paid advertisements on Facebook, Instagram, and Twitter, emails to the CREST.BD mailing list and healthcare providers or organizations associated with the CREST.BD network, and featured at online (webinar) and in-person educational events (held in Vancouver, Canada) for individuals with BD. To address initial overrepresentation of white North American participants, social media advertisements specifically targeted users in Asia, Africa, and South America with interests related to BD (i.e., liking social media pages for the International Bipolar Foundation, BP Magazine for Bipolar, and the Depression and Bipolar Support Alliance). Participants were entered into a prize draw for one of two $50 (CAD) Visa gift cards. Inclusion criteria were: (1) age 19 or older, and (2) a self-reported diagnosis of BD.

## Measures

An online survey was developed on the basis of previous literature and stakeholder input (Additional file 1). Individuals were asked to describe their use of apps to track or support their symptoms and quality of life across a range of domains identified as important to people with BD (Michalak and Murray 2010).

Given concern about the type and quality of apps used to support symptom management in BD (Nicholas et al. 2015; Lagan et al. 2020a), the present analysis focused on items concerning the use of apps related to the core foci of BD self-management (i.e., mood and sleep). Individuals were asked if they used apps to a) track or manage their mood or b) support their sleep/wake routine or sleep quality. If a respondent indicated yes, they were asked to name the apps used and describe their frequency of use. Evaluation of apps used to support other life domains was beyond scope of this analysis.

### Data analysis

#### Use of self-management apps to support mood or sleep

Descriptive statistics were used to characterize the sample and summarize the use of smartphones and self-management apps. One author (LL) categorized free-text responses indicating names of apps used; internet searches were used to confirm the spelling and existence of apps referenced.

#### Evaluation of commonly used self-management apps

The five most commonly nominated mood and sleep self-management apps were subject to further analysis. This cutoff was selected to ensure the evaluation process was feasible and swift, given concerns regarding the currency of app evaluation papers (Larsen et al. 2020), and was chosen for its similarity to the number of apps reported by prior studies of the most frequently named apps used by people with depression/anxiety (Rubanovich et al. 2017), and a survey of health apps used by the general population (Krebs and Duncan 2015).

#### The MIND framework

To support comparability with a recent review of apps identified by marketplace searches of the term “bipolar” (Lagan et al. 2020a), we used the Mhealth Index and Navigation Database (MIND) framework (Lagan et al. 2020b) based on the American Psychiatric Association’s (APA) app evaluation model. The initial APA framework was developed through consultation with service users, clinicians, and informaticists (American Psychiatric Association 2017), and amalgamated questions from 45 existing evaluation frameworks (Henson et al. 2019). The MIND framework consists of 105 objective questions that map onto the APA framework and principles of medical ethics, and is divided into six sections: app origin and functionality (background information and accessibility), inputs and outputs (what types of data are collected and generated), privacy and security (data use and sharing, patient safety), clinical foundation (the existence of peer reviewed literature describing the feasibility and efficacy of reviewed apps), features and engagement style (app interface and overall functionality), interoperability and data sharing (ability to export/share data for personal or clinical use). The MIND framework has been used to describe the quality and safety of mental health apps for schizophrenia (Lagan et al. 2021a), bipolar disorder (Lagan et al. 2020a), Spanish language speakers (Muñoz et al. 2021), peripartum affective disorders (Feldman et al. 2021), and top-returned apps for mental health related searches (Lagan et al. 2021b).

It should be noted that as the MIND framework is intended for use by non-specialists (including people with lived experience, healthcare administrators, and policymakers) to provide a *general* assessment of the quality of a given app, a formal systematic review is not required to answer items in the clinical foundation section, nor are raters instructed in evaluating the quality of scientific literature (Lagan et al. 2021b). In a similar vein, the MIND framework generates a *descriptive* summary of app characteristics; no total score or quantitative summary is provided. In practice, clinicians are encouraged to conduct a personalized assessment of the fit between a reviewed app and an individual’s needs/priorities (Torous et al. 2018).

#### Rater training

All raters (LL, MC, RK, LY) underwent publicly available training in the framework questions (apps.digitalpsych.org). Following training, raters applied the framework questions to two apps that were not the subject of the present investigation (7Cups and ACT iCoach) to ensure reliability with expert-generated criterion ratings. Discrepancies were addressed via discussion with the framework developers. All raters met the minimum threshold for very good interrater reliability, defined as Cohen’s kappa statistic > 0.75 (McHugh 2012), and percentage agreement with criterion ratings was high (> 80%).

#### Evaluation procedures in the present study

Each app was assessed by at least two raters. Raters used their personal devices to download and test apps. All apps were evaluated between January and April, 2021.

Raters identified peer-reviewed studies by searching Google Scholar and Medline using the app name and “feasibility”, “efficacy” or “effectiveness” as keywords. The reference lists of identified studies were also reviewed for additional relevant literature. To support the feasibility and relevance of the literature review, efficacy studies describing unrelated physical health outcomes (e.g., weight, caloric intake, physical activity) were excluded. This decision was made following a preliminary search for literature related to the Fitbit app, in which numerous studies related to other functions of this app and associated wearable device were identified.

Discrepancies between raters were resolved via consensus discussion. Descriptive statistics were used to summarize key findings from the resulting data.

## Results

### Survey sample

A total of 919 people with BD responded to the online survey (see Table 1 for demographic details). 81.3% of participants completed the survey between June 21 and July 20. Participants were primarily female (77.9%, *n* = 716), white (61%, *n* = 560), residing in North America (43.2%, *n* = 397), with a mean age of 36.9 years (SD = 12). Approximately half the sample self-reported a BD-II diagnosis (51.9%, *n* = 477), and most participants were receiving psychiatric treatment, including medication (81.1%, *n* = 745) and counselling (47.7%, *n* = 438). The sample was largely employed (55.3%, *n* = 508), married or in a committed relationship (49.5%, *n* = 455), and had completed some form of post-secondary education (77.4%, *n* = 711). Almost all participants reported using apps on a smartphone (97.5%, *n* = 896).

**Table 1:** Sample characteristics

Demographic variable	Total	Mood/sleep app use (*n* = 382)	No mood/sleep app use (*n* = 537)
Sex (female); *n* (%)	716 (77.9%)	308 (80.6%)	408 (76%)
Age; mean (SD)	36.9 (12)	36.9 (12)	37 (12)
BD subtype; *n* (%)			
BD I BD II Other/no formal diagnosis	321 (34.9%) 477 (51.9%) 121 (13.2%)	139 (36.4%) 198 (51.8%) 45 (11.8%)	182 (33.9%) 279 (52%) 76 (14.1%)
Current treatment; *n* (%)			
Medication Counselling/psychotherapy Peer support	745 (81.1%) 438 (47.7%) 142 (15.5%)	318 (83.2%) 212 (55.5%) 67 (17.5%)	427 (79.5%) 226 (42.1%) 75 (14%)
Ethnicity; *n* (%)			
White Black/African Asian Middle Eastern Latin American Other or multiple ethnicities	560 (61%) 40 (4.4%) 152 (16.6%) 22 (2.4%) 48 (5.2%) 96 (10.5%)	251 (65.7%) 15 (3.9%) 47 (12.3%) 8 (2.1%) 19 (5%) 42 (11%)	309 (57.6%) 25 (4.7%) 105 (19.6%) 14 (2.6%) 29 (5.4%) 54 (10.1%)
Country of residence; *n* (%)			
Canada United States of America Europe Australia/New Zealand Asia Middle East Africa Latin America	169 (18.4%) 228 (24.8%) 105 (11.4%) 37 (4%) 160 (17.4%) 19 (2.1%) 142 (15.5%) 56 (6.1%)	88 (23%) 108 (28.3%) 47 (12.3%) 20 (5.2%) 48 (12.6%) 8 (2.1%) 37 (9.7%) 25 (6.5%)	81 (15.1%) 120 (22.3%) 58 (10.8%) 17 (3.2%) 112 (20.9%) 11 (2%) 105 (19.6%) 31 (5.8%)
Education level; *n* (%)			
Any high school Postsecondary Undergraduate Postgraduate Other	177 (19.3%) 214 (23.3%) 324 (35.3%) 173 (18.8%) 31 (3.4%)	56 (14.7%) 83 (21.7%) 157 (41.1%) 75 (19.6%) 11 (2.9%)	121 (22.5%) 131 (24.4%) 167 (31.1%) 98 (18.2%) 20 (3.7%)
Employment status; *n* (%)			
Working full-time Working part-time Self-employed Student Not in paid employment Retired	341 (37.1%) 156 (17%) 11 (1.2%) 102 (11.1%) 263 (28.6%) 46 (5%)	140 (36.6%) 75 (19.6%) 1 (0.3%) 41 (10.7%) 106 (27.7%) 19 (5%)	201 (37.4%) 81 (15.1%) 10 (1.9%) 61 (11.4%) 157 (29.2%) 27 (5%)
Marital status; *n* (%)			
Single Married/in a committed relationship Divorced/separated Other/prefer not to say	347 (37.8%) 455 (49.5%) 96 (10.4%) 21 (2.3%)	143 (37.4%) 193 (50.5%) 40 (10.5%) 6 (1.6%)	204 (38%) 262 (48.8%) 56 (10.4%) 15 (2.8%)
Devices used to access apps			
Smartphone Tablet Neither	896 (97.5%) 209 (22.7%) 10 (1.1%)	379 (99.2%) 107 (28%) 1 (0.2%)	517 (96.3%) 102 (19%) 9 (1.7%)

### Use of self-management apps to support mood or sleep

In total, 41.6% (*n* = 382) of the sample reported using a self-management app related to mood and/or sleep. Specifically, 24.8% (*n* = 228) of respondents reported use of an app to monitor or improve mood, and 26.3% (*n* = 242) reported use of an app to monitor or improve sleep (see Table 2). Of individuals using self-management apps, 23% (*n* = 88) reported using apps related to both mood and sleep. Daily use of self-management apps was common.

**Table 2:** Use of BD-related apps

Use of apps	Total; *n* (%)
Use of mood-related self-management apps	228 (24.8%)
Frequency of use:	
More than once a day Daily Weekly or less often	60 (26.3%) 99 (43.4%) 69 (30.3%)
Use of sleep-related self-management apps	242 (26.3%)
Frequency of use:	
More than once a day Daily Weekly or less often	21 (8.8%) 156 (65.3%) 62 (25.9%)

A broad spectrum of apps were nominated in relation to each self-management domain. A total of 110 unique apps were described as being used to monitor or improve mood, however the majority were nominated only once (68.2%, *n* = 75). Similarly, while 104 apps were named in relation to monitoring or promoting optimal sleep, most sleep apps were nominated only once (73.1%; *n* = 76).

### Evaluation of commonly used self-management apps

The top five apps related to mood were nominated a total of 130 times, with the most popular mood app (Daylio) nominated by 59 respondents. Other commonly nominated mood apps included Bipolar eMoods Tracker (*n* = 45), iMood Journal (*n* = 9), Moodpath (*n* = 9), and Calm (*n* = 8). The top five sleep-related apps were nominated a total of 112 times, with the most popular app (Fitbit) mentioned 50 times. Other commonly nominated sleep apps included Calm (*n* = 24), Sleep Cycle (*n* = 15), Samsung Health (*n* = 14), and Headspace (*n* = 9). 25 people reported using the alarm function of the pre-installed clock app to support their sleep—as this a function of the smartphone itself, we chose to exclude it from further analysis.

As one app (Calm) appeared in the top five most commonly nominated apps in relation to both mood and sleep self-management, a final set of nine apps was evaluated using the MIND framework. The summary below highlights trends across the reviewed apps. For a full description of reviewed apps according to the 105 framework questions, see Additional file 2. To provide readers with an accessible, brief overview of findings, key characteristics of the nine reviewed apps are also presented in Table 3.

**Table 3:** Brief summary of key characteristics of the most commonly nominated mood and sleep self-management apps (n = 9)

	Daylio Journal	eMoods Bipolar Mood Tracker	iMood Journal (Mood Diary)	MoodPath Depression and Anxiety Tester	Calm	Headspace	Fitbit	Sleep Cycle	Samsung Health
Survey nominations	Mood; *n* = 59	Mood; *n* = 45	Mood; *n* = 9	Mood; *n* = 9	Mood; *n* = 8 Sleep; *n* = 24	Sleep; *n* = 9 Mood; *n* = 4	Sleep; *n* = 50	Sleep; *n* = 15	Sleep; *n* = 14
Cost	In-app purchases	In-app purchases	Upfront cost	In-app purchases	In-app purchases	In-app purchases	In-app purchases	In-app purchases	Free
Health information	Not shared	Not shared	Not shared	Not shared	Not shared	Shared with third parties	Not shared	Shared with third parties	Shared with third parties
Can users delete data?	Yes	No	No	Yes	Yes	No	Yes	Yes	No
Crisis support	No	Yes	No	Yes	No	No	No	No	No
Feasibility/ efficacy research	1 feasibility study	1 feasibility study	No	1 efficacy study	3 feasibility studies, 6 efficacy studies	13 feasibility studies, 20 efficacy studies	3 feasibility studies, 32 efficacy studies	1 feasibility study	3 efficacy studies
Data export	Yes	Yes	Yes	Yes	Yes	No	Yes	Yes	Yes
Key features	Mood, sleep, and exercise tracking	Mood, medication, and sleep tracking	Mood, medication, and sleep tracking	Mood tracking, psycho-education, mindfulness	Mood tracking, mindfulness, relaxation, sleep therapy	Psycho-education, mindfulness, relaxation, sleep therapy	Sleep/activity tracking, psycho-education, mindfulness, relaxation	Mood and sleep tracking, mindfulness	Sleep and exercise tracking

#### Origin and accessibility

At the time of this analysis, all reviewed mood and sleep apps were available on both the Apple iOS and Google Play stores, and had been updated in the last 180 days—a metric associated with user satisfaction and safety (Wisniewski et al. 2019). Reviewed apps appeared to be popular, with an average rating of 4.6/5 on the iOS store. All reviewed apps were commercial; no developers were affiliated with university, healthcare, or government organizations. All but one app was free to download, although the majority (*n* = 8) required in-app purchases to access all content. Only a small proportion of apps (*n* = 2) required a continuous internet connection. All apps were available in a language other than English, and the majority (*n* = 6) had one or more accessibility features (e.g., adjustable text size or text-to-voice).

#### Inputs and outputs

Reviewed mood and sleep self-management apps most commonly collected user-input data via surveys (*n* = 7) and diaries (*n* = 7). Few (*n* = 3) received data from external devices (e.g., wearables). App outputs typically included notifications (*n* = 9) or graphs of data (*n* = 8); few had the option to share information to the user’s social network (*n* = 4) or contained a link to formal care or coaching (*n* = 1).

#### Privacy and confidentiality

All reviewed apps had a publicly available privacy policy that described data use and purpose (*n* = 9), and the majority (*n* = 7) described security measures in place. Few (*n* = 3) apps shared personal health information (e.g., name, birthday, mental health information); data were more commonly shared with third parties if deidentified (i.e., stripped of personally identifiable attributes; *n* = 8) or anonymized (i.e., no traceable link to an individual, *n* = 8). User control over data was limited: four apps allowed users to opt out of data collection, and five apps allowed users to delete their data. The accessibility of privacy policies to the average user was poor: the average Flesch-Kincaid grade-reading level of the privacy policies was 13.45, suggesting that a high-school education or some post-secondary education would be required to read and understand the content. Concerningly, only two apps contained crisis management features (e.g., a number for a suicide prevention hotline).

#### Clinical foundation

Raters did not identify any potentially harmful content in the reviewed apps. The majority of mood and sleep self-management apps (*n* = 6) had published evidence regarding their feasibility/usability, largely in healthy adults (summarized in Additional file 3). Few apps nominated in relation to mood (*n* = 2) had evidence to support their efficacy, compared to apps nominated in relation to sleep (*n* = 4). Existing efficacy studies provided some support for developers’ claims that app use could result in improved mindfulness, stress management, sleep quality, and mood; the design, sample, and findings of these studies are summarized in Additional file 4. However, only one efficacy study included a BD sample.

#### Features, engagement style, and data sharing

Apps nominated in relation to mood typically included the following features: mood tracking (*n* = 5), sleep tracking (*n* = 3), and journaling (*n* = 5). Apps nominated in relation to sleep typically included the following features: sleep tracking (*n* = 3), journaling (*n* = 3), mindfulness (*n* = 4) and deep breathing (*n* = 3) exercises, and physical health exercises (*n* = 3). Reviewed self-management apps were typically designed for self-monitoring rather than the delivery of psychosocial interventions: no mood app was observed to offer elements of cognitive behavior therapy, acceptance and commitment therapy, or dialectical behavior therapy. Two sleep apps were observed to offer elements of cognitive behavior therapy for insomnia.

To support engagement, the majority of both types of self-management apps incorporated audiovisual elements (*n* = 6), and all incorporated user-generated data (*n* = 9). However, peer support (*n* = 1) and gamification (*n* = 4) were rarely offered. Most apps (*n* = 8) allowed users to email or export their data, although only three were designed for use with a clinician in conjunction with a treatment plan.

## Discussion

The present study used a large-scale, international web-based survey to identify and evaluate apps used to support mood and sleep, two key foci of self-management in BD. Use of health and wellbeing apps was moderately prevalent: 42% of the sample reported using an app to support self-management of either mood or sleep. These individuals reported a high frequency of mHealth use, suggesting apps were perceived as helpful and engaging. Interestingly, although a number of apps offered both mood and sleep tracking, the reviewed apps were generally nominated in relation to one purpose only. Only Calm and Headspace were nominated in relation to both sleep and mood. The analysis of the quality, safety, and efficacy of the top five sleep and mood self-management apps nominated by people with BD (for a total of nine reviewed apps) offers some reassuring findings, as well as some cautions.

A body of evidence supports the feasibility and acceptability of included apps (Additional file 3), largely in healthy adults. However, extrapolation of this data is limited by the short-term nature of these studies, with the longest follow-up period being eight weeks. Additionally, the presence of reminders from the research team could encourage app uptake beyond what might be expected in a naturalistic setting (Baumel et al. 2019). Individuals with BD may engage, disengage, and re-engage with apps according to the fluctuating course of illness (O'Brien et al. 2020). Although one BD-specific app (eMoods Bipolar Mood Tracker) was evaluated, this was conducted with a sample of healthy volunteers (Tena-Cucala and Cobo 2019). Only one qualitative study of the Headspace app included people with a BD diagnosis (Mistler et al. 2017). Thus, these studies may not be an accurate reflection of the likelihood of engagement in a non-study setting for those living with mental illness. While participants in the present study were asked about their frequency of app use, they were not asked about the length of time since app download. Research on mHealth apps more broadly suggests that sustained engagement in real-world contexts is rare (Fleming et al. 2018). Naturalistic, long-term research in this population is therefore needed to better determine app usage patterns, as available research regarding adherence to apps developed for BD suggests highly heterogenous rates of use (Patoz et al. 2021). Feasibility research is also necessary to identify and address accessibility barriers unique to people with BD, who may experience cognitive difficulties (Bonnín et al. 2021), hand tremors (Gitlin 2016), and fluctuations in attention and motivation in accordance with mood state (Urošević et al. 2008), that together influence app usage.

Evidence for the efficacy of apps is varied (Additional file 4). While the impacts of some apps (i.e., Headspace and Fitbit) have been extensively researched, others had no or little peer reviewed evidence (i.e., Daylio Journal, eMoods Bipolar Mood Tracker, iMood Journal, MoodPath and Sleep Cycle). Although a formal systematic review of the literature and evaluation of study quality was outside scope of this analysis (nor is it required as part of the MIND framework app evaluation), it is worth noting broad trends in the identified research: while some randomized control trials had reasonably large samples, many others had an inactive control, used a single-arm design, and had small sample sizes. Further, the use of healthy volunteers may introduce a sampling bias, as those who have an interest in mHealth may be more likely to engage in these studies. Finally, while some of the outcomes assessed are directly relevant to BD (e.g., mood and sleep quality), many of the studies measured variables related to general wellbeing, such as mindfulness, stress, and attention, and only one qualitative study included people with BD (Mistler et al. 2017). Further research is required to more confidently state the potential efficacy of such app-based interventions in BD populations. In addition, the possibility of harm resulting from use of these apps in people with BD, such as the potential of symptom monitoring exacerbating negative affect, is also not known (Nicholas et al. 2019; Faurholt-Jepsen et al. 2015).

Given widespread concerns related to the privacy and data security of health and mental health apps (Robillard et al. 2019; Huckvale et al. 2019), it is notable that the apps most often used by individuals with BD all had an available privacy policy describing data use and purpose. However, the high reading grade of the policies echoed a commonly cited issue, that the readability and comprehension of policies are often inaccessible (Robillard et al. 2019). Although this raises obvious questions concerning *informed* consent, trust, and transparency of health apps, calls for more user-friendly privacy information have gained little traction. Further, user control over data among these widely used apps was lacking: under half the apps assessed enabled user control over the collection or deletion of data, and most apps shared data with third parties. Research has shown that individuals with mental health difficulties have varied feelings about sharing data, depending on the recipient and type of data shared (Nicholas et al. 2019). However, allowing users to have nuanced control over data collection, sharing, and retention is often at odds with how commercially developed apps make their profits. Collecting, analyzing, and selling personal data is central to the new digital economy. Even deidentified data may be interpreted and recombined in ways that potentially reveal sensitive information, such as routine visits to a psychiatrist’s office, or engaging with mental health content on social media. It is therefore important that healthcare providers are aware of ethical issues related to the functioning of the digital economy (Bauer et al. 2017), in order to discuss the benefits and risks of app use with patients. In addition, further efforts on behalf of app store platforms are needed, including regulation of health app developers and demands for transparency regarding data use.

In comparison to published reviews of BD app evidence-base and privacy, the current findings are encouraging. Whilst reviews of the broader BD app landscape report very low incidence of scientific evidence for available apps (Larsen et al. 2019), 55% of the apps most commonly used by individuals with BD have published evidence (albeit, with the limitations discussed above). In terms of privacy, reviews in the area have reported that the provision of privacy policies among apps for BD increased from 22% in 2015 (Nicholas et al. 2015), to 68% in 2020 (Lagan et al. 2020a). The nine most popular mood and sleep self-management apps reviewed herein each had a privacy policy. These commonly used apps also perform well compared to a review of privacy policies for mental health tracking apps (Robillard et al. 2019), which reported only 18% of iOS and 4% of Android apps made privacy policies available. Comparative to reviews of the general app landscape regarding evidence-base and privacy, the present analysis of commonly reviewed apps suggests that many people with BD are self-selecting apps that are of reasonable quality. However, it should be cautioned that only a small percentage of reviewed apps offered crisis support information for distressed users.

Notably, the most popular mood and sleep apps used by people with BD are not condition-specific, rather, they are ranked among the most prominent health and wellbeing apps for the general population. For example, Fitbit is one of the most commonly installed health and fitness apps amongst US mobile owners (Krebs and Duncan 2015); similarly, Calm, Headspace, and Daylio rank among the most commonly downloaded mental health apps (Carlo et al. 2019; Woodward et al. 2020). While the apps most frequently nominated in this survey were found to largely meet minimum standards for safety and privacy, previous reviews have noted limitations regarding adherence to clinical guidelines for both mood and sleep apps (Nicholas et al. 2015; Lagan et al. 2020a; Grigsby-Toussaint et al. 2017; Qu et al. 2020), and their efficacy for improving outcomes in a BD population is unknown. Importantly, generic health apps may recommend strategies with the potential for harm in a BD population, or fail to detect early warning signs unique to this group. For example, sleep restriction is a technique that can be used to address insomnia in the general population, however this should be used with caution in BD due to the potential for triggering mania (Morton and Murray 2020). Similarly, unidimensional self-report mood scales (i.e., sad to happy) may inappropriately reinforce problematic mood elevation in BD. Apps designed for the general public or unipolar depression may offer suggestions to increase activity in response to low mood; people with BD should be warned that such strategies, while helpful, can trigger or exacerbate manic symptoms. In the absence of widely adopted, evidence-supported, condition-specific apps for BD, clinicians play an important role in supporting patients to recognize and work around the limitations of generic health and wellbeing apps.

Although use of specific apps by people with BD has not previously been investigated, a survey of physical and mental health apps used by people with depression and anxiety found similar trends (Rubanovich et al. 2017). This sample (*n* = 176) was specifically recruited to test the IntelliCare suite of mental health apps, and as such, findings may not have been reflective of the population of mental health app users more generally. Even so, some similarities with the present analysis were observed: the most commonly used health apps included ones designed for the general population, such as Fitbit, Samsung Health, and Headspace. Of note, of the 139 unique health apps mentioned, 92.8% had less than 5 mentions, and 69.8% had only one mention. This accords with the present study, where 68.2% of named mood apps and 73.1% of named sleep apps were nominated by only one person. Analysis of the rate of download of health apps more broadly suggests that the majority of apps are downloaded less than 5000 times (Aitken and Lyle 2015). Together, these findings highlight a large degree of interindividual variability in the precise apps used to manage health concerns. As such, while the present findings offer some confidence in regards to the quality and safety of the most commonly used apps, clinicians should still be alert to (and enquiring of) the possibility that patients are using poorer quality apps (Aref-Adib et al. 2020).

The preponderance of generic health and wellbeing apps used by respondents in the present study (and the large number of people reporting daily use of such health apps) raises a number of considerations for the development and dissemination of mHealth interventions for BD. Firstly, it is necessary to weigh whether user priorities for aesthetics and functionality are being sufficiently addressed in BD-specific apps: indeed, a qualitative analysis of public reviews of apps developed for BD highlighted a number of unmet needs and complaints regarding functionality (Nicholas et al. 2017a), suggesting that consumer preferences are currently not adequately addressed. This emphasizes the need for research-led app development projects to solicit potential user perspectives during the design phase, as clinicians/researchers and the public may not share priorities for content, features, or aesthetics (Carlo et al. 2019). Secondly, this finding highlights the need for researchers to consider scale-up and commercialization as part of end-of-grant planning (Hidalgo-Mazzei et al. 2020). More research must be conducted into effective dissemination strategies for evidence-informed mHealth interventions. People with mental health conditions (including those in the present study) primarily rely on word of mouth or app store ratings/reviews to identify apps (Rubanovich et al. 2017; Morton et al. 2022; Schueller et al. 2018), which may have contributed to the popularity of generic health and wellbeing apps seen here. Training initiatives may be required to support people with BD in identifying and selecting the most appropriate apps for their specific health needs (Hoffman et al. 2020). Such efforts should be complementary to training and education for healthcare providers; although they may be a valuable source of information on apps, a survey found that only 50% of clinicians have discussed or suggested apps to patients with BD (Morton et al. 2021b). Clinicians who had not discussed apps commonly cited lack of knowledge regarding safe and appropriate apps for this population as a barrier. Finally, the potential negatives of condition-specific apps also require further consideration; there is evidence that symptom monitoring in BD can be experienced as an unpleasant reminder of illness (Bendegem et al. 2014), and potential app users may also be concerned about unintended disclosure through titles and icons that contain references to the diagnosis (Nicholas et al. 2017b). Ideally, awareness raising could encourage developers of popular health apps to include content tailored for this mood disorder, much in the way that generic stress management apps often contain some psychoeducation about clinical depression or anxiety. Indeed, new initiatives targeting transdiagnostic symptoms in psychiatric disorders, in which intervention content is tailored to daily assessments, have shown positive results (Ben-Zeev et al. 2018).

### Strengths and limitations

A number of limitations to the present study should be noted. Firstly, our sample was predominantly female, white, employed, and had completed some form of post-secondary education. As such, findings may not generalize to individuals with BD more broadly. Further, estimates of app use may be higher amongst respondents to a web-based survey relative to community samples, although rates of smartphone ownership are similar to other large-scale BD surveys (Hidalgo-Mazzei et al. 2019). Our survey findings may also be reflective of increased uptake of digital health tools associated with the COVID-19 pandemic (Sorkin et al. 2021). Participants were informed that a BD diagnosis was required to be eligible for the survey, and were given the option to self-report a BD subtype. As diagnosis was not confirmed with a clinical interview, it is possible that individuals who did not meet diagnostic criteria for BD may have completed the survey, and findings should be interpreted with requisite caution. Although there is limited research on the characteristics of individuals who self-identify as having BD, one study reassuringly supports that people who self-identify as having BD typically meet diagnostic criteria (Kupfer et al. 2002): a random sample of people who registered to join a BD care registry (*n* = 100) found that 93% met DSM-IV criteria for lifetime BD following a face-to-face structured clinical interview. In addition, rates of survey completion were likely enhanced by not including a requirement to complete a clinical interview, which would have added significantly to response burden, and detracted from study feasibility (in terms of staff, budget, and time required). On balance, a strength of our study is the large sample size and international representation, given that app use may vary by region (Woodward et al. 2020), with 56.5% of the sample residing outside of North America.

Secondly, there is no consensus approach to evaluating mHeath apps (Torous et al. 2018). A recent scoping review found the MIND categories and questions largely overlap with content encompassed in other existing app evaluation frameworks, suggesting good coherence (Lagan et al. 2021); however, some aspects of user experience (e.g., usability and aesthetic appeal) are not addressed. It has been suggested that the subjectivity inherent in rating such aspects is a likely source of inconsistencies in app evaluations (Carlo et al. 2019). Given this, a strength of using the MIND framework is that it focuses on objective criteria; use of this framework also permits comparison between our analysis of user- nominated apps and earlier work on marketplace offerings for BD (Lagan et al. 2020a).

Our evaluation of apps identified in the survey was not intended as an endorsement of any specific apps for clinical purposes; rather we intended to provide a snapshot of the quality, safety, and efficacy of apps commonly used by people with BD. As such, we chose to focus on apps used to support two core foci of self-management in BD (mood and sleep). Self-management in BD encompasses a broad range of strategies (Murray et al. 2011), and the present analysis may not address all potential targets of apps in BD, such as crisis management/safety planning, medication reminders, fitness and nutrition, amongst others (Murnane et al. 2016). Although we asked participants to describe their use of apps related to other QoL domains, these were not subject to further formal analysis for reasons of feasibility. Additionally, the MIND evaluation framework was developed to describe mental health apps – there may be specific risks, privacy standards, or features which should be described to comprehensively evaluate apps designed for other purposes (e.g., social networking, financial management). However, a strength of this focused approach means our review was less likely to be impacted by the high turnover of marketplace offerings identified as problematic in previous publications (Larsen et al. 2020); indeed, we note that at the time of submission, all reviewed apps remained available in the Google Play and Apple app marketplaces. Finally, although we assessed privacy policies of the included apps, data protections and use as written does not necessarily reflect true practices (Huckvale et al. 2019).

## Conclusion

Taken together, these findings reinforce the importance of contextualizing app evaluations according to real-world usage rates, through demonstrating that the earlier reviews of the BD app landscape are not necessarily reflective of apps most commonly used in real-world contexts. Rather, people with BD tended to use apps designed for the general population to support and monitor their mood and sleep. The presence of some privacy protections and evidence regarding app efficacy in healthy adults offers some reassuring findings in regards to quality of apps used by people with BD. However, there is a paucity of evidence exploring benefits and harms of use of these apps specific to BD which requires further consideration. Research-led mHealth development projects should consider how user-centered design and effective dissemination plans may increase the reach and uptake of apps.

## Supplementary Information

**Additional file 1.** Survey of App Use: Perspectives from people living with bipolar disorder.

**Additional file 2.** Evaluation of the most commonly nominated mood and sleep self-management apps (n = 9) using the MIND framework.

**Additional file 3: Table S3. **List of studies evaluating the feasibility of the most commonly nominated mood and sleep self-management apps (n = 9).

**Additional file 4: Table S2. **List of studies evaluating the efficacy or validity of the most commonly nominated mood and sleep self-management apps (n = 9).

## Data Availability

Data are not publicly available in accordance with ethics approval given by the ethics board from the participating university. Interested investigators may submit inquiries to the corresponding author.

## Abbreviations

BD: Bipolar disorder
CREST.BD: Collaborative RESearch Team to study psychosocial issues in Bipolar disorder
