# Supplementary material for: Evaluating the quality, safety, and functionality of commonly used smartphone apps for bipolar disorder mood and sleep self-management
Source: Int J Bipolar Disord. 2022 Apr 4;10:10. doi: 10.1186/s40345-022-00256-6 (PMC8977125; doi:10.1186/s40345-022-00256-6)
Supplement: Supplementary file 3 — Additional file 3: Table S3. List of studies evaluating the feasibility of the most commonly nominated mood and sleep self-management apps (n = 9). [file 40345_2022_256_MOESM3_ESM.docx]

*Supplementary Table 3.* List of studies evaluating the feasibility of highly endorsed mood and sleep self-management apps.

| **Author** | **App** | **Sample** | **Study design** | **Outcome measures** | **Findings** |
| --- | --- | --- | --- | --- | --- |
| Hussain et al. (2020) | Daylio | Healthy adults (*n* = 14) | Cross-sectional study | Task completion, completion time, error rates | Participants were able to complete tasks on the app quickly and with few mistakes. Older participants and those on iOS had greater difficulty with task completion. |
| Tena-Cucala et al. (2019) | eMoods | Healthy adults (*n* = 30) | Cross-sectional study | Usefulness, Satisfaction and Ease of Use Questionnaire, Computer System Usability Questionnaire | Participants described the app as intuitive, quick and easy to use, with some limitations in interface and presentation. |
| Avalos et al. (2020) | Headspace | Women with postpartum depression  (*n* = 27) | 6-week single-arm study | Semi structured interviews, retention and adherence rates | 74% of participants used the app at least once. 33% used the app over 50% of days during the intervention period. 69% reported being very or extremely satisfied with the app and found it easy and convenient to use. |
| Champion et al. (2018) | Headspace | Healthy adults  (*n* = 74) | 30-day RCT, waitlist control | Engagement and experience questionnaire, app usage patterns | Most participants indicated the app was neither easy nor hard to use and rated enjoyment >4 on a scale out of 7. Participants used the app an average of 6 days during the first ten days. |
| Costalupes et al. (2021) | Headspace | High school baseball players (*n* = 4) | 2-week pilot study | Author-designed survey | Participants found the app was enjoyable, convenient, fun and effective to use. |
| Kashat et al. (2020) | Headspace | Otolaryngology residents  (*n* = 8) | 6-week pilot study | Satisfaction survey using Likert scale | Participants rated app quality 3.71/5, and most expressed interest in further engaging with a mindfulness practice. |
| Hunter et al. (2019) | Headspace | Mothers with children with Fragile X  (*n* = 18) | Mixed methods pilot study | App usage patterns, semi-structured interviews | Program with app use had a 72% completion rate. Those who did not complete the program cited forgetfulness or not enjoying program. |
| Laurie et al. (2016) | Headspace | Healthy adults  (*n* = 16) | 30-40 day single-arm study | Qualitative interviews | Barriers to use included lack of routine, strong negative emotions and negative perceptions of mindfulness. Enabling factors were positive perceptions of app/mindfulness, realistic expectations and positive social influences. |
| McGuire et al. (2019) | Headspace | Pharmacy students  (*n* = 23) | Single semester pilot study | Qualitative questions on app experience | Most participants reported that the app was easy and enjoyable to use. Barriers including forgetfulness, lack of time and challenging techniques. |
| Mistler et al. (2017) | Headspace | Inpatients with schizophrenia, schizoaffective or bipolar disorder  (*n* = 13) | 1 week single-arm study | Qualitative interviews | All participants reported finding the app easy to use, understand and learn. 10 participants used the app for all 7 days of the intervention. |
| Noone et al. (2018) | Headspace | Healthy university students  (*n* = 91) | 6-week RCT, active control (guided breathing app) | Objective adherence data, Technology Acceptance Model questionnaire, binary questions on intervention acceptability | Participants reported finding app to be enjoyable and easy to use. On average, 15 sessions were completed. |
| Rosen et al. (2018) | Headspace | Women diagnosed with breast cancer  (*n* = 112) | 8-week RCT (12 week follow-up), waitlist control | App log data, self-reported app utilization and study completion | Participants used the app on average 18 days during the intervention period with a retention rate of 67%. Uptake was higher in those with higher baseline quality of life. |
| Taylor et al. (2016) | Headspace | Pediatric residents  (*n* = 33) | 10-day pilot study | Survey on perceptions of mindfulness practice | 11/33 participants completed the study. Barriers to use included lack of time, lack of knowledge, forgetfulness. |
| Wen et al. (2017) | Headspace | Surgical residents  (*n* = 43) | 4-week pilot study | App usage patterns, app rating on Likert scale | 70% of participants were able to adhere to the study. Participants rated app usefulness 2.86/4. |
| Zollars et al. (2019) | Headspace | Pharmacy students  (*n* = 92) | 4-week quasi-experimental study | Self-report adherence log | 64/65 participants completed >75% of the suggested meditation time, 54 completed 100%. Participants indicated they were likely to continue meditating after study was complete. |
| Huberty, Eckert et al. (2019) | Calm | Patients with myeloproliferative neoplasm  (*n* = 128) | 4-group cross-over RCT (8 weeks), active control (psychoeducation or alternative meditation app) | Investigator-developed survey related to app use and satisfaction, adherence to intervention | 55% of participants used the app at least 5 times per week. 79% reported enjoying app content. |
| Huberty, Green et al. (2019) | Calm | College students with elevated stress (*n* = 88) | 8-week RCT (12-week follow-up), waitlist control | Satisfaction survey, adherence to intervention | 85% of participants reported enjoying app use; 85% reported satisfaction with the app. >50% indicated they would continue use in future and recommend it to others. App use was on average 38 minutes/week. |
| Huberty et al. (2020) | Calm | Adult cancer patients  (*n* = 82) | Cross-sectional survey | Investigator-developed satisfaction survey | 83% of participants enjoyed using the app, 84% were satisfied with content and 97% would recommend it to others. Participants used the app an average of 71 minutes/week. |
| Castner et al. (2019) | Fitbit | Females with poorly controlled asthma  (*n* = 44) | Cross-sectional study | Percentage of data available, number of consecutive nights of device wear | Participants wore the Fitbit for an average of 16.43 consecutive nights, with 12.4% of data missing (due to device removal, damage/loss, dead battery, activity/restlessness, and participants skipping a night). |
| Scott et al. (2019) | Fitbit | Teenagers accessing youth mental health services (*n* = 13) | Cross-sectional study | Verbal feedback on acceptability | Participants indicated finding the Fitbit to be highly acceptable, and preferred its use over the Actigraph as it did not feel like a medical device and allowed for real-time data access. |
| Visovsky et al. (2013) | Fitbit | Healthy females (*n* = 3) | Cross-sectional study | Focus group assessing device experience | Participants found Fitbit and Actigraph to be easy to use and acceptable. They preferred the Fitbit due to features such as notifications, graphic feedback and ability to log food/water consumption. |

**References**

1. Hussain A, Mkpojiogu E, Hussein I, et al. The effectiveness, efficiency and reliability-in-use of Daylio mobile app. *Int J Adv Sci Technol*. 2020;29:180-187.

2. Tena-Cucala R, Cobo J. Usabilidad, satisfacción y facilidad de uso de aplicaciones (apps) gratuitas en español para pacientes con trastorno bipolar / Usability, satisfaction and ease of use of free apps in spanish for patients with bipolar disorder. *Psicosomática y Psiquiatría*. 2019;2019:13-38.

3. Avalos LA, Aghaee S, Kurtovich E, et al. A mobile health mindfulness intervention for women with moderate to moderately severe postpartum depressive symptoms: Feasibility study. *JMIR Ment Health.* 2020;7(11):e17405.

https://doi.org/10.2196/17405

4. Champion L, Economides M, Chandler C. The efficacy of a brief app-based mindfulness intervention on psychosocial outcomes in healthy adults: A pilot randomised controlled trial. *PLoS One.* 2018;13(12):e0209482. https://doi.org/10.1371/journal.pone.0209482

5. Costalupes B, Gilbert JN, Gilbert W, et al. A smartphone mindfulness-based intervention pilot study with competitive high school baseball players. *Journal of Kinesiology and Wellness*. 2020;9:63-72.

6. Kashat L, Carter B, Mosha M, et al. Mindfulness education for otolaryngology residents: A pilot study. *OTO Open.* 2020;4(3):2473974X20945277-22473974X20945277. https://doi.org/10.1177/2473974X20945277

7. Hunter JE, Jenkins CL, Grim V, et al. Feasibility of an app-based mindfulness intervention among women with an FMR1 premutation experiencing maternal stress. *Res Dev Disabil.* 2019;89:76-82. https://doi.org/10.1016/j.ridd.2019.03.008

8. Laurie J, Blandford A. Making time for mindfulness. *Int J Med Inform.* 2016;96. https://doi.org/10.1016/j.ijmedinf.2016.02.010

9. McGuire J, Zhen TJIip. Use of a mindfulness smartphone app in an advanced psychiatry elective for pharmacy students*. Innov Pharm*. 2019;10(3):13-13. https://doi.org/10.24926/iip.v10i3.1346

10. Mistler LA, Ben-Zeev D, Carpenter-Song E, et al. Mobile mindfulness intervention on an acute psychiatric unit: Feasibility and acceptability study. *JMIR Ment Health.* 2017;4(3):e34. https://doi.org/10.2196/mental.7717

11. Noone C, Hogan MJ. A randomised active-controlled trial to examine the effects of an online mindfulness intervention on executive control, critical thinking and key thinking dispositions in a university student sample. *BMC Psychol.* 2018;6(1):13. https://doi.org/10.1186/s40359-018-0226-3

12. Rosen KD, Paniagua SM, Kazanis W, et al. Quality of life among women diagnosed with breast cancer: A randomized waitlist controlled trial of commercially available mobile app-delivered mindfulness training. *Psychooncology.* 2018;27(8):2023-2030. https://doi.org/10.1002/pon.4764

13. Taylor M, Hageman JR, Brown M. A mindfulness intervention for residents: Relevance for pediatricians. *Pediatr Ann.* 2016;45(10):e373-e376. https://doi.org/10.3928/19382359-20160912-01

14. Wen L, Sweeney TE, Welton L, et al. Encouraging mindfulness in medical house staff via smartphone app: A pilot study. *Acad Psychiatry.* 2017;41(5):646-650. https://doi.org/10.1007/s40596-017-0768-3

15. Zollars I, Poirier TI, Pailden J. Effects of mindfulness meditation on mindfulness, mental well-being, and perceived stress. *Curr Pharm Teach Learn.* 2019;11(10):1022-1028. https://doi.org/10.1016/j.cptl.2019.06.005

16. Huberty J, Eckert R, Larkey L, et al. Smartphone-based meditation for myeloproliferative neoplasm patients: Feasibility study to inform future trials. *JMIR Form Res.* 2019;3(2):e12662. https://doi.org/10.2196/12662

17. Huberty J, Green J, Glissmann C, et al. Efficacy of the mindfulness meditation mobile app "calm" to reduce stress among college students: Randomized controlled trial. *JMIR mHealth and uHealth.* 2019;7(6):e14273-e14273. https://doi.org/10.2196/14273

18. Huberty J, Puzia M, Eckert R, et al. Cancer patients’ and survivors’ perceptions of the calm app: Cross-sectional descriptive study. *JMIR Cancer*. 2020;6(1):e16926. https://doi.org/10.2196/16926

19. Castner J, Mammen MJ, Jungquist CR, et al. Validation of fitness tracker for sleep measures in women with asthma. *J Asthma.* 2019;56(7):719-730. https://doi.org/10.1080/02770903.2018.1490753

20. Scott J, Grierson A, Gehue L, et al. Can consumer grade activity devices replace research grade actiwatches in youth mental health settings? *Sleep Biol Rhythms.* 2019;17(2):223-232. https://doi.org/10.1007/s41105-018-00204-x

21. Visovsky C, Kip KE, Rice JL, et al. Choosing instruments for research: An evaluation of two activity monitors in healthy women. *J Nov Physiother*. 2013; 3(5):171. https://doi.org/10.4172/2165-7025.1000171
