# Supplementary material for: Evaluating the quality, safety, and functionality of commonly used smartphone apps for bipolar disorder mood and sleep self-management
Source: Int J Bipolar Disord. 2022 Apr 4;10:10. doi: 10.1186/s40345-022-00256-6 (PMC8977125; doi:10.1186/s40345-022-00256-6)
Supplement: Supplementary file 4 — Additional file 4: Table S2. List of studies evaluating the efficacy or validity of the most commonly nominated mood and sleep self-management apps (n = 9). [file 40345_2022_256_MOESM4_ESM.docx]

*Supplementary Table 2.* List of studies evaluating the efficacy or validity of highly endorsed mood and sleep self-management apps.

| **Author** | **App** | **Sample** | **Study design** | **Outcome measures** | **Findings** |
| --- | --- | --- | --- | --- | --- |
| Burchert et al. (2021) | Moodpath | Convenience sample of app users  (*n* = 113) | 14-day pilot study | PHQ-9 | Moodpath was psychometrically similar to the PHQ-9 with high sensitivity and good specificity. |
| Economides et al. (2018) | Headspace | Healthy adults  (*n* = 69) | 1-month RCT with active control (psychoeducation audiobook) | Stress Overload Scale, Scale of Positive and Negative Experience, Brief Irritability Test | Intervention group experienced reduction in irritability and stress, and improvement in affect. |
| Flett et al. (2020) | Headspace | University students  (*n* = 250) | 6-month RCT, waitlist control | Kessler Psychological Distress Scale, College Adjustment Test, Brief Resilience Scale, New General Self-Efficacy Scale, Cognitive Affective Mindfulness Scale, academic scores | Intervention group had small improvements in college adjustment, mindfulness, and affect, and no improvement in resilience or self-efficacy. |
| Bostock et al. (2019) | Headspace | Healthy employees at a UK company  (*n* = 238) | 8-week RCT (16-week follow-up), waitlist control | Warwick Edinburgh Mental Well-being Scale, Hospital Anxiety and Depression Scale, Whitehall II Study Questionnaire, Freiburg Mindfulness Inventory, workplace support, blood pressure | Intervention group experienced improved well-being, positive affect, distress, job strain, workplace support and decreased blood pressure. |
| Flett et al (2019) | Headspace | University students  (*n* = 208) | 10-day RCT (30-day follow-up), placebo control (note-taking app) | Centre for Epidemiological Studies Depression Scale, Hospital Anxiety and Depression Scale-Anxiety Subscale, Perceived Stress Scale, Brief Resilience Scale, Flourishing Scale, College Adjustment Test, Cognitive Affective Mindfulness Scale-Revised | Intervention group experienced improvement in depressive symptoms, college adjustment, resilience and mindfulness. There was no improvement in flourishing. |
| Bennike et al. (2017) | Headspace | Healthy adults  (*n* = 137) | 30 day RCT, placebo control (brain-training app) | Sustained Attention to Response Task, Mindful Attention to Awareness Scale | Headspace group experienced decreased mind wandering and increased dispositional mindfulness. |
| Morrison Wylde et al. (2017) | Headspace | Pediatric nursing residents  (*n* = 95) | 4-week RCT (3 month follow-up), active control (face-to-face mindfulness training) | Compassion Fatigue Self Test, Life Events Checklist, Posttraumatic stress disorder Checklist-Civilian Version, Five Facet Mindfulness Questionnaire | Headspace group experienced more “acting with awareness” and non-reactivity skills, and less compassion fatigue and burnout. |
| Yang et al. (2018) | Headspace | Medical students  (*n* = 88) | 30-day RCT (60-day follow-up), waitlist control | Perceived Stress Scale, Five Facet Mindfulness Questionnaire, General Well-being Schedule | Intervention group experienced decreased perceived stress and increased well-being. |
| Howells et al. (2016) | Headspace | “Happiness seekers”  (*n* = 121) | 10-day RCT, placebo control (checklist app) | Satisfaction with Life Scale, Flourishing Scale, Positive and Negative Affect Scale, Center for Epidemiologic Studies Depression Scale | Intervention group experienced increases in positive affect and reduced depressive symptoms. No group differences in satisfaction, flourishing or negative affect. |
| Noone et al. (2018) | Headspace | University students  (*n* = 91) | 6-week RCT, active control (guided breathing app) | Halpern Critical Thinking Assessment, Heuristics and Biases items, Five Facet Mindfulness Questionnaire, Sternberg Working Memory Task, Positive and Negative Affect Schedule, Warwick-Edinburgh Mental Wellbeing Scale, Real World Outcomes Inventory | Both groups experienced an increase in mindfulness disposition and critical thinking scores. No significant difference in outcomes between groups. |
| Laurie et al. (2016) | Headspace | Healthy adults  (*n* = 16) | 30-40 day single-arm study | Qualitative interviews | Participants reported feeling calm and relaxed after use. |
| Mistler et al. (2017) | Headspace | Inpatients with schizophrenia, schizoaffective or bipolar disorder  (*n* = 13) | 1 week single-arm study | Qualitative interviews | Participants reported increased focus and ability to fall asleep, decreased anger and anxiety, and improved mood. |
| Champion et al. (2018) | Headspace | Healthy adults  (*n* = 74) | 30-day RCT, waitlist control | General Health Questionnaire 28, Satisfaction with Life Scale, Perceived Stress Scale, Wagnild Resilience Scale, engagement and experience questionnaire | Intervention group reported positive impacts on life satisfaction, stress and resilience at day 10 with further improvements at day 30. |
| Rosen et al. (2018) | Headspace | Women diagnosed with breast cancer  (*n* = 112) | 8-week RCT (12-week follow-up), waitlist control | Functional Assessment of Cancer Therapy-Breast version, Mindful Attention Awareness Scale | Intervention group reported higher quality of life and dispositional mindfulness. |
| Zollars et al. (2019) | Headspace | Pharmacy students  (*n* = 92) | 4-week quasi-experimental study | Five Facet Mindfulness Questionnaire, Warwick-Edinburgh Mental Well-Being Scale, Perceived Stress Scale | Participants experienced increased mindfulness, mental well-being and decreased perceived stress following Headspace use. |
| McGuire et al. (2019) | Headspace | Pharmacy students  (*n* = 23) | Single semester pilot study | Mindfulness Attention Awareness Scale | No statistically significant change in mindfulness however the students subjectively reported improvements in their wellbeing and stress levels. |
| Costalupes et al. (2021) | Headspace | High school baseball players (*n* = 4) | 2-week pilot study | Author-designed survey | Participants indicated an understanding of mindfulness concepts, improved relaxation during stressful situations and increased self-confidence after Headspace use. |
| Kashat et al. (2020) | Headspace | Otolaryngology residents  (*n* = 8) | 6-week pilot study | Mindful Attention Awareness Scale, Positive and Negative Affect Schedule | Participants reported a decrease in negative affect, which was positively correlated with mindfulness scores. |
| Wen et al. (2017) | Headspace | Surgical residents  (*n* = 43) | 4-week pilot study | Positive and Negative Affect Schedule, Freiburg Mindfulness Inventory | There was an increase in mindfulness and a trend towards an increase in positive affect. No changes in negative affect. |
| Taylor et al. (2016) | Headspace | Pediatric residents  (*n* = 33) | 10-day pilot study | Maslach Burnout Inventory, Mindful Attention Awareness Scale | No statistically significant changes were observed. However, participants were more likely to perceive mindfulness as a useful intervention after the study. |
| Avalos et al. (2020) | Headspace | Women with postpartum depression  (*n* = 27) | 6-week single-arm study | PHQ-8, Perceived Stress Scale, Pittsburgh Sleep Quality Index, Five Facet Mindfulness Questionnaire | Participants experienced significant improvements in depression, stress, sleep quality and mindfulness. |
| Huberty, Vranceanu et al. (2019) | Calm | Calm subscribers  (*n* = 12,151) | Cross-sectional survey | Survey developed by the research team regarding changes in mental health, physical health, stress and sleep after Calm use. | Sleep stories use was associated with changes in sleep and physical health. Meditation use was associated with changes in mental and physical health and stress. |
| Clarke et al. (2020) | Calm | University students  (*n* = 269) | 7day single-arm study | Five Facet Mindfulness Questionnaire, Generalized Self-Efficacy Scale, Short Warwick-Edinburgh Mental Well-being Scale | Participants noted improvements in wellbeing, trait mindfulness and self-efficacy. Qualitative data suggests some experienced increased anxiety and lowered self-efficacy. |
| Luberto et al. (2021) | Calm | Calm subscribers with cardiovascular disease  (*n* = 1512) | Cross-sectional survey | Survey developed by the research team regarding engagement with the app, clinical characteristics and changes in mental health, physical health, stress and sleep after Calm use. | Most participants reported changes in sleep, stress, physical and mental health following app use. These changes were more marked in those with pre-existing anxiety and depression. |
| Huberty, Eckert et al. (2019) | Calm | Patients with myeloproliferative neoplasm  (*n* = 128) | 4-group cross-over RCT (8 weeks), active control (psychoeducation or alternative meditation app) | Satisfaction survey, National Institutes of Health Patient Reported Outcomes Measurement Information System, MPN Symptom Assessment Form | Participants who used Calm experienced significant improvement in sleep and physical health and non-significant effects on anxiety, depression, total symptom burden and fatigue. |
| Huberty et al. (2020) | Calm | Adult cancer patients  (*n* = 82) | Cross-sectional survey | Survey developed by research team regarding Calm use. | Participants reported Calm use reduced reactivity, increased emotional awareness, present moment attention, and increased sense of peace and gratitude. |
| Huberty, Green et al. (2019) | Calm | College students with elevated stress (*n* = 88) | 8-week RCT (12-week follow-up), waitlist control | Perceived Stress Scale, Five Factor Mindfulness Questionnaire, Self-Compassion Survey Short-Form, health behaviours, Patient-Reported Outcomes Measurement Information System, Youth Risk Behaviour Surveillance | Most participants found intervention helpful in reducing their stress. Intervention did not impact physical health outcomes. |
| Xie et al. (2018) | Samsung Health | University students  (*n* = 44) | Cross-sectional study | Heart rate, number of steps, energy consumption, sleep duration | Mean absolute percentage error for the Samsung Gear S3 for sleep duration was 0.06 +/- 0.10. Of devices assessed for sleep, Samsung had the best performance. |
| Kubala et al. (2020) | Samsung Health | Healthy adults  (*n* = 20) | Cross-sectional study | Pittsburgh Sleep Diary, Actiwatch sleep monitoring | Samsung and Actiwatch did not have significantly different total sleep time, the mean absolute percent error was 5.1%. Samsung underestimated wake after sleep onset by an average of 15.9 minutes, compared to Actiwatch. |
| Asgari Mehrabadi et al. (2020) | Samsung Health | Healthy adults  (*n* = 45) | Cross-sectional study | Actigraphy | Samsung Gear overestimated total sleep time by an average of 22.51 min, overestimated sleep efficiency by 4.44% and underestimated wake after sleep onset by 31.27 min. |
| Beattie et al. (2017) | Fitbit | Healthy adults (*n* = 60) | Cross-sectional study | Type III home sleep testing device, including EEG channels | Sleep staging reached a per-epoch accuracy of 69%, with misclassifications of light/REM or light/wake. There was no under or overestimation of sleep duration. |
| Brooke et al. (2017) | Fitbit | Healthy adults (*n* *=* 95) | Cross-sectional study | Sleep log | Mean absolute percent errors for Fitbit Flex when compared to a sleep log were 8.8% and 11.5% for Fitbit Charge HR which fell within the equivalence zone. |
| Castner et al. (2019) | Fitbit | Females with poorly controlled asthma  (*n* = 44) | Cross-sectional survey | Actigraphy | Fitbit Charge had a 97% sensitivity and 40% specificity in identification of sleep. There was good equivalence and concordance in detection of wake counts, total sleep time and sleep efficiency. |
| Cook et al. (2017) | Fitbit | Adults with unipolar major depressive disorder  (*n* =21) | Cross-sectional study | Polysomnography and actigraphy | Fitbit Flex overestimated sleep time and efficiency and did not accurately identify wake epochs in a free-living setting. |
| Cook et al. (2019) | Fitbit | Patients with suspected central disorders of hypersomnolence (*n* = 49) | Cross-sectional study | Polysomnography | Fitbit Alta HR overestimated total sleep time, sleep efficiency and total sleep duration. It was unable to detect sleep-onset rapid eye movement periods. |
| de Zambotti et al. (2016) | Fitbit | Healthy adolescents  (*n* = 32) | Cross-sectional study | Polysomnography | Fitbit Charge HR demonstrated good accuracy and sensitivity in detecting sleep but low specificity in detecting waking. There was tendency towards overestimation of total sleep time, sleep efficiency and underestimation of wake after sleep onset. |
| de Zambotti et al. (2018) | Fitbit | Healthy adults (*n =* 44) | Cross-sectional study | Polysomnography | Fitbit Charge 2 overestimated total sleep time and underestimated sleep onset latency. Time spent in REM and wake after sleep onset were accurately measured. |
| Dickinson et al. (2016) | Fitbit | Healthy adults (*n* = 38) | Cross-sectional study | Actigraphy | Fitbit devices overestimated sleep and demonstrated less sensitivity than actigraphy. |
| Ferguson et al. (2015) | Fitbit | Healthy adults (*n =* 21) | Cross-sectional study | Actigraphy | Fitbit One correlated well with actigraphy in measuring sleep duration, with r = 0.92. |
| Godino et al. (2020) | Fitbit | Healthy children aged 9-11 (*n* = 26) | Cross-sectional study | Polysomnography | Fitbit Charge HR had a 95.8% sensitivity in classifying sleep but a 56.3% specificity in detecting wake. It tended to underestimate total sleep time and overestimate wake after sleep onset. |
| Haghayegh et al. (2020) | Fitbit | Healthy adults (*n* = 35) | Cross-sectional study | Actigraphy and EEG | Fitbit Charge 2 underestimated sleep onset latency and overestimated sleep efficiency. There was good agreement in wake after sleep onset, total sleep time and detection of REM and deep sleep. |
| Hakim et al. (2018) | Fitbit | Children with sleep disordered breathing  (*n* = 22) | Cross-sectional study | Polysomnography | Fitbit Charge overestimated total sleep time and underestimated total wake time, with limited overall concordance. |
| Kang et al. (2017) | Fitbit | Healthy adults (*n* = 21) and those with insomnia disorder  (*n* = 41) | Cross-sectional study | Polysomnography | Fitbit Flex was well correlated with polysomnography for total sleep time, with a tendency for overestimation. Agreement was lower in the insomnia group than for good sleepers. |
| Kubala et al. (2020) | Fitbit | Healthy adults (*n* = 30) | Cross-sectional study | Actigraphy | Fitbit Alta underestimated wake after sleep onset and overestimated total sleep time. Mean absolute percent error was 36.6%. Results were similar between good and poor sleepers. |
| Lee et al. (2017) | Fitbit | Healthy adults (*n* = 16) | Cross-sectional study | Actigraphy | Fitbit Charge HR was highly correlated with actigraphy, with a tendency to overestimate sleep durations. |
| Lee et al. (2018) | Fitbit | Healthy adults (*n* = 78) | Cross-sectional study | Sleep diary | Fitbit Charge HR had a mean absolute percentage error 14.2% for total sleep time and of 12.7% for time in bed. |
| Liang et al. (2018) | Fitbit | Healthy adults (*n* = 25) | Cross-sectional study | Medical sleep monitor | Fitbit Charge 2 was accurate in detecting onset and offset of sleep, with comparable results in sleep efficiency and total sleep duration as the medical monitor. Measures of sleep structure were poor. |
| Liang et al. (2019) | Fitbit | Healthy adults (*n* = 23) | Cross-sectional study | Medical sleep monitor | Fitbit Charge 2 underestimated sleep stage transitions and was significantly different from medical monitor in detecting these changes. |
| Liu et al. (2019) | Fitbit | Healthy adults (*n* =10) | Cross-sectional study | Consensus sleep diary | There was a large variance between Fitbit Alta and the sleep diary, particularly with awakenings during the night. |
| Mantua et al. (2016) | Fitbit | Healthy adults (*n* = 40) | Cross-sectional study | Polysomnography | Fitbit Flex correlated strongly with polysomnography for total sleep however not for sleep efficiency, light sleep time and deep sleep time. |
| Maskevich et al. (2017) | Fitbit | Adults with Huntington’s Disease (*n* = 7) | Cross-sectional study | Polysomnography | Fitbit One overestimated total sleep time and sleep efficiency and was not sufficiently accurate. |
| Meltzer et al. (2015) | Fitbit | Healthy youth aged 3-17  (*n =* 63) | Cross-sectional study | Polysomnography and actigraphy | Fitbit Ultra was sensitive and accurate however not specific in measuring total sleep time and sleep time, with a tendency to overestimate. |
| Montgomery-Downs et al. (2012) | Fitbit | Healthy adults (*n* = 24) | Cross-sectional study | Polysomnography and actigraphy | Fitbit was significantly different from actigraphy and polysomnography on total sleep time and sleep efficiency, with a tendency to overestimate. |
| Moreno-Pino et al. (2019) | Fitbit | Adults with obstructive sleep apnea  (*n =* 65) | Cross-sectional study | Polysomnography | Fitbit was significantly different from polysomnography on all measures except rapid eye movement sleep, with overestimation of total sleep time and underestimation of wake after sleep onset and sleep onset latency. |
| Osterbauer et al. (2016) | Fitbit | Children with sleep-disordered breathing  (*n* = 14) | Cross-sectional study | Polysomnography | Fitbit was sensitive for sleep detection but not specific for wake detection. |
| Rosenberger et al. (2016) | Fitbit | Healthy adults (*n* = 40) | Cross-sectional study | Z-machine | Fitbit One overestimated total sleep time and was not specific in detecting sleep/wake. |
| Sargent et al. (2018) | Fitbit | Young athletes (*n =* 12) | Cross-sectional study | Polysomnography | Fitbit HR Charge overestimated total sleep time and poorly detected daytime naps. |
| Scott et al. (2019) | Fitbit | Teenagers accessing youth mental health services (*n* = 13) | Cross-sectional study | Actigraphy | Fitbit overestimated total sleep time by an hour and underestimated wake after sleep onset by half an hour. |
| Stone et al. (2020) | Fitbit | Healthy adults (*n* = 5) | Cross-sectional study | EEG-based device | Fitbit was highly accurate in measuring total sleep time, total wake time and sleep efficiency. |
| Svensson et al. (2019) | Fitbit | Healthy adults (*n* = 20) | Cross-sectional study | Portable EEG system | Fitbit Versa correlated well with EEG for time in bed, total sleep time and sleep efficiency with a 92.1% sensitivity and 54.1% specificity. Significant differences existed in identification of specific sleep stages, sleep efficiency, sleep onset latency, sleep period time and wake after sleep onset. |
| Tedesco et al. (2019) | Fitbit | Healthy adults (*n* = 20) | Cross-sectional study | Actigraphy | Fitbit Charge 2 slightly overestimated total sleep time with a mean absolute percentage error of 10.13%. Estimation of wake after sleep onset was poor, with a tendency to overestimation. |
| Visovsky et al. (2013) | Fitbit | Healthy females (*n* = 3) | Cross-sectional study | Actigraphy | Fitbit had limited reliability in assessing total sleep time, number of nighttime awakenings and sleep efficiency. Concordance correlation coefficients ranged from 0.66-0.70. |

**References**

1. Burchert S, Kerber A, Zimmermann J, et al. Screening accuracy of a 14-day smartphone ambulatory assessment of depression symptoms and mood dynamics in a general population sample: Comparison with the PHQ-9 depression screening. *PLoS One.* 2021;16(1):e0244955. https://doi.org/10.1371/journal.pone.0244955

2. Economides M, Martman J, Bell MJ, et al. Improvements in stress, affect, and irritability following brief use of a mindfulness-based smartphone app: A randomized controlled trial. *Mindfulness (N Y).* 2018;9(5):1584-1593. https://doi.org/10.1007/s12671-018-0905-4

3. Flett JAM, Conner TS, Riordan BC, et al. App-based mindfulness meditation for psychological distress and adjustment to college in incoming university students: A pragmatic, randomised, waitlist-controlled trial. *Psychol Health.* 2020;35(9):1049-1074. https://doi.org/10.1080/08870446.2019.1711089

4. Bostock S, Crosswell AD, Prather AA, et al. Mindfulness on-the-go: Effects of a mindfulness meditation app on work stress and well-being. *J Occup Health Psychol.* 2019;24(1):127-138. https://doi.org/10.1037/ocp0000118

5. Flett JAM, Hayne H, Riordan BC, Thompson LM, Conner TS. Mobile mindfulness meditation: A randomised controlled trial of the effect of two popular apps on mental health. *Mindfulness.* 2019;10(5):863-876.

6. Bennike IH, Wieghorst A, Kirk U. Online-based mindfulness training reduces behavioral markers of mind wandering. *J Cogn Enhanc.* 2017;1(2):172-181. https://doi.org/10.1007/s41465-017-0020-9

7. Morrison Wylde C, Mahrer NE, Meyer RML, et al. Mindfulness for novice pediatric nurses: Smartphone application versus traditional intervention. *J Pediatr Nurs.* 2017;36:205-212. https://doi.org/10.1016/j.pedn.2017.06.008

8. Yang E, Schamber E, Meyer RML, et al. Happier healers: Randomized controlled trial of mobile mindfulness for stress management. *J Altern Complement Med.* 2018;24(5):505-513. https://doi.org/10.1089/acm.2015.0301

9. Howells A, Ivtzan I, Eiroa-Orosa FJ. Putting the ‘app’ in happiness: A randomised controlled trial of a smartphone-based mindfulness intervention to enhance wellbeing. *J Happiness Stud.* 2016;17(1):163-185. https://doi.org/10.1007/s10902-014-9589-1

10. Noone C, Hogan MJ. A randomised active-controlled trial to examine the effects of an online mindfulness intervention on executive control, critical thinking and key thinking dispositions in a university student sample. *BMC Psychol.* 2018;6(1):13. https://doi.org/10.1186/s40359-018-0226-3

11. Laurie J, Blandford A. Making time for mindfulness. *Int J Med Inform.* 2016;96:38-50. https://doi.org/10.1016/j.ijmedinf.2016.02.010

12. Mistler LA, Ben-Zeev D, Carpenter-Song E, et al. Mobile mindfulness intervention on an acute psychiatric unit: Feasibility and acceptability study. *JMIR Ment Health.* 2017;4(3):e34. https://doi.org/10.2196/mental.7717

13. Champion L, Economides M, Chandler C. The efficacy of a brief app-based mindfulness intervention on psychosocial outcomes in healthy adults: A pilot randomised controlled trial. *PLoS One.* 2018;13(12):e0209482. https://doi.org/10.1371/journal.pone.0209482

14. Rosen KD, Paniagua SM, Kazanis W, et al. Quality of life among women diagnosed with breast cancer: A randomized waitlist controlled trial of commercially available mobile app-delivered mindfulness training. *Psychooncology.* 2018;27(8):2023-2030. https://doi.org/10.1002/pon.4764

15. Zollars I, Poirier TI, Pailden J. Effects of mindfulness meditation on mindfulness, mental well-being, and perceived stress. *Curr Pharm Teach Learn.* 2019;11(10):1022-1028. https://doi.org/10.1016/j.cptl.2019.06.005

16. McGuire J, Zhen TJIip. Use of a mindfulness smartphone app in an advanced psychiatry elective for pharmacy students. *Innov Pharm*. 2019;10(3):13-13. https://doi.org/[10.24926/iip.v10i3.1346](https://dx.doi.org/10.24926%2Fiip.v10i3.1346)

17. Costalupes B, Gilbert JN, Gilbert W, et al. A smartphone mindfulness-based intervention pilot study with competitive high school baseball players. 2020;9:63-72.

18. Kashat L, Carter B, Mosha M, et al. Mindfulness education for otolaryngology residents: A pilot study. *OTO Open.* 2020;4(3):2473974X20945277-22473974X20945277. https://doi.org/[10.1177/2473974X20945277](https://dx.doi.org/10.1177%2F2473974X20945277)

19. Wen L, Sweeney TE, Welton L, et al. Encouraging mindfulness in medical house staff via smartphone app: A pilot study. *Acad psychiatry.* 2017;41(5):646-650. https://doi.org/10.1007/s40596-017-0768-3

20. Taylor M, Hageman JR, Brown M. A mindfulness intervention for residents: Relevance for pediatricians. *Pediatr Ann.* 2016;45(10):e373-e376. https://doi.org/10.3928/19382359-20160912-01

21. Avalos LA, Aghaee S, Kurtovich E, et al. A mobile health mindfulness intervention for women with moderate to moderately severe postpartum depressive symptoms: Feasibility study. *JMIR Ment Health.* 2020;7(11):e17405.

https://doi.org/10.2196/17405

22. Huberty J, Vranceanu AM, Carney C, et al. Characteristics and usage patterns among 12,151 paid subscribers of the Calm meditation app: Cross-sectional survey. *JMIR Mhealth Uhealth.* 2019;7(11):e15648. https://doi.org/10.2196/15648

23. Clarke J, Draper S. Intermittent mindfulness practice can be beneficial, and daily practice can be harmful. An in depth, mixed methods study of the “Calm” app's (mostly positive) effects. *Internet Interv.* 2020;19:100293. https://doi.org/10.1016/j.invent.2019.100293

24. Luberto CM, Huberty J, Puzia M, et al. Usage patterns of the Calm meditation app among people with cardiovascular disease. *Mindfulness.* 2021;12(4):983-993. https://doi.org/10.1007/s12671-020-01567-4

25. Huberty J, Eckert R, Larkey L, et al. Smartphone-based meditation for myeloproliferative neoplasm patients: Feasibility study to inform future trials. *JMIR Form Res.* 2019;3(2):e12662. https://doi.org/10.2196/12662

26. Huberty J, Puzia M, Eckert R, et al. Cancer patients’ and survivors’ perceptions of the calm app: Cross-sectional descriptive study. *JMIR Cancer*. 2020;6(1):e16926. https://doi.org/10.2196/16926

27. Huberty J, Green J, Glissmann C, et al. Efficacy of the mindfulness meditation mobile app "calm" to reduce stress among college students: Randomized controlled trial. *JMIR mHealth and uHealth.* 2019;7(6):e14273-e14273. https://doi.org/10.2196/14273

28. Xie J, Wen D, Liang L, et al. Evaluating the validity of current mainstream wearable devices in fitness tracking under various physical activities: Comparative study. *JMIR Mhealth Uhealth.* 2018;6(4):e94. https://doi.org/10.2196/mhealth.9754

29. Kubala AG, Barone Gibbs B, Buysse DJ, et al. Field-based measurement of sleep: Agreement between six commercial activity monitors and a validated accelerometer. *Behav Sleep Med.* 2020;18(5):637-652. https://doi.org/10.1080/15402002.2019.1651316

30. Asgari Mehrabadi M, Azimi I, Sarhaddi F, et al. Sleep tracking of a commercially available smart ring and smartwatch against medical-grade actigraphy in everyday settings: Instrument validation study. *JMIR Mhealth Uhealth.* 2020;8(10):e20465. https://doi.org/10.2196/20465

31. Beattie Z, Oyang Y, Statan A, et al. Estimation of sleep stages in a healthy adult population from optical plethysmography and accelerometer signals. *Physiol Meas.* 2017;38(11):1968-1979. https://doi.org/10.1088/1361-6579/aa9047

32. Brooke SM, An HS, Kang SK, et al. Concurrent validity of wearable activity trackers under free-living conditions. *J Strength Cond Res.* 2017;31(4):1097-1106. https://doi.org/10.1519/JSC.0000000000001571

33. Castner J, Mammen MJ, Jungquist CR, et al. Validation of fitness tracker for sleep measures in women with asthma. *J Asthma.* 2019;56(7):719-730. https://doi.org/10.1080/02770903.2018.1490753

34. Cook JD, Prairie ML, Plante DT. Utility of the Fitbit Flex to evaluate sleep in major depressive disorder: A comparison against polysomnography and wrist-worn actigraphy. *J Affect Disord.* 2017;217:299-305. https://doi.org 10.1016/j.jad.2017.04.030

35. Cook JD, Eftekari SC, Dallmann E, et al. Ability of the Fitbit Alta HR to quantify and classify sleep in patients with suspected central disorders of hypersomnolence: A comparison against polysomnography. *J Sleep Res.* 2019;28(4):e12789. https://doi.org/10.1111/jsr.12789

36. de Zambotti M, Baker FC, Willoughby AR, et al. Measures of sleep and cardiac functioning during sleep using a multi-sensory commercially-available wristband in adolescents. *Physiol Behav.* 2016;158:143-149. https://doi.org/10.1016/j.physbeh.2016.03.006

37. de Zambotti M, Goldstone A, Claudatos S, et al. A validation study of Fitbit Charge 2™ compared with polysomnography in adults. *Chronobiol Int.* 2018;35(4):465-476. https://doi.org/10.1080/07420528.2017.1413578

38. Dickinson DL, Cazier J, Cech T. A practical validation study of a commercial accelerometer using good and poor sleepers. *Health Psychol Open.* 2016;3(2):2055102916679012. https://doi.org 10.1177/2055102916679012

39. Ferguson T, Rowlands AV, Olds T, et al. The validity of consumer-level, activity monitors in healthy adults worn in free-living conditions: a cross-sectional study*. Int J Behav Nutr Phys Act*. 2015;12(1):1-9. https://doi.org/10.1186/s12966-015-0201-9

40. Godino JG, Wing D, de Zambotti M, et al. Performance of a commercial multi-sensor wearable (Fitbit Charge HR) in measuring physical activity and sleep in healthy children. *PLoS One.* 2020;15(9):e0237719. https://doi.org/10.1371/journal.pone.0237719

41. Haghayegh S, Khoshnevis S, Smolensky MH, et al. Performance assessment of new-generation Fitbit technology in deriving sleep parameters and stages. *Chronobiol Int.* 2020;37(1):47-59. https://doi.org/10.1080/07420528.2019.1682006

42. Hakim M, Miller R, Hakim M, et al. Comparison of the Fitbit® Charge and polysomnography for measuring sleep quality in children with sleep disordered breathing. *Minerva Pediatr.* 2018. https://doi.org/10.23736/S0026-4946.18.05333-1

43. Kang SG, Kang JM, Ko KP, et al. Validity of a commercial wearable sleep tracker in adult insomnia disorder patients and good sleepers. *J Psychosom Res.* 2017;97:38-44. https://doi.org/10.1016/j.jpsychores.2017.03.009

44. Lee HA, Lee HJ, Moon JH, et al. Comparison of wearable activity tracker with actigraphy for sleep evaluation and circadian rest-activity rhythm measurement in healthy young adults. *Psychiatry Investig.* 2017;14(2):179-185. https://doi.org/10.4306/pi.2017.14.2.179

45. Lee JM, Byun W, Keill A, et al. Comparison of wearable trackers' ability to estimate sleep. *Int J Environ Res Public Health.* 2018;15(6):1265. https://doi.org/10.3390/ijerph15061265

46. Liang Z, Chapa Martell MA. Validity of consumer activity wristbands and wearable EEG for measuring overall sleep parameters and sleep structure in free-living conditions. *J Healthc Inform Res.* 2018;2(1):152-178. https://doi.org/10.1007/s41666-018-0013-1

47. Liang Z, Chapa-Martell MA. Accuracy of Fitbit wristbands in measuring sleep stage transitions and the effect of user-specific factors. *JMIR mHealth and uHealth.* 2019;7(6):e13384-e13384. https://doi.org/10.2196/13384

48. Liu J, Wong WT, Zwetsloot IM, et al. Preliminary agreement on tracking sleep between a wrist-worn device Fitbit Alta and consensus sleep diary. *Telemed J E Health.* 2019;25(12):1189-1197. https://doi.org/10.1089/tmj.2018.0202

49. Mantua J, Gravel N, Spencer RM. Reliability of sleep measures from four personal health monitoring devices compared to research-based actigraphy and polysomnography. *Sensors (Basel).* 2016;16(5). https://doi.org/10.3390/s16050646

50. Maskevich S, Jumabhoy R, Dao PDM, et al. Pilot validation of ambulatory activity monitors for sleep measurement in Huntington's Disease gene carriers. *J Huntingtons Dis.* 2017;6(3):249-253. https://doi.org/10.3233/JHD-170251

51. Meltzer LJ, Hiruma LS, Avis K, et al. Comparison of a commercial accelerometer with polysomnography and actigraphy in children and adolescents. *Sleep.* 2015;38(8):1323-1330. https://doi.org/10.5665/sleep.4918

52. Montgomery-Downs HE, Insana SP, Bond JA. Movement toward a novel activity monitoring device. *Sleep Breath*. 2012;16(3):913-917. https://doi.org/10.1007/s11325-011-0585-y

53. Moreno-Pino F, Porras-Segovia A, López-Esteban P, et al. Validation of Fitbit Charge 2 and Fitbit Alta HR against polysomnography for assessing sleep in adults with obstructive sleep apnea. *J Clin Sleep Med.* 2019;15(11):1645-1653. https://doi.org/10.5664/jcsm.8032

54. Osterbauer B, Koempel J, Ward S, et al. A comparison study of the Fitbit activity monitor and PSG For assessing sleep patterns and movement in children. *Otolaryngol.* 2016;1(3):24-25. https://doi.org/10.14302/issn.2379-8572.joa-15-891

55. Rosenberger ME, Buman MP, Haskell WL, et al. Twenty-four hours of sleep, sedentary behavior, and physical activity with nine wearable devices. *Med Sci Sports Exerc.* 2016;48(3):457-465. https://doi.org/10.1249/MSS.0000000000000778

56. Sargent C, Lastella M, Romyn G, et al. How well does a commercially available wearable device measure sleep in young athletes? *Chronobiol Int.* 2018;35(6):754-758. https://doi.org/10.1080/07420528.2018.1466800

57. Scott J, Grierson A, Gehue L, et al. Can consumer grade activity devices replace research grade actiwatches in youth mental health settings? *Sleep Biol Rhythms.* 2019;17(2):223-232. https://doi.org/10.1007/s41105-018-00204-x

58. Stone JD, Rentz LE, Forsey J, et al. Evaluations of commercial sleep technologies for objective monitoring during routine sleeping conditions. *Nat Sci Sleep.* 2020;12:821-842. https://doi.org/10.2147/NSS.S270705

59. Svensson T, Chung UI, Tokuno S, et al. A validation study of a consumer wearable sleep tracker compared to a portable EEG system in naturalistic conditions. *J Psychosom Res.* 2019;126:109822. https://doi.org/10.1016/j.jpsychores.2019.109822

60. Tedesco S, Sica M, Ancillao A, et al. Validity evaluation of the Fitbit Charge2 and the Garmin Vivosmart HR+ in free-Living environments in an older adult cohort. *JMIR Mhealth Uhealth*. 2019;7(6):e13084. https://doi.org/10.2196/13084

61. Visovsky C, Kip KE, Rice JL, et al. Choosing instruments for research: An evaluation of two activity monitors in healthy women. *J Nov Physiother.* 2013; 3(5):171. https://doi.org/10.4172/2165-7025.1000171
